# Environmentally Sensitive Molecular Switches Drive Poplar Phenology

**DOI:** 10.3389/fpls.2018.01873

**Published:** 2018-12-17

**Authors:** Jay P. Maurya, Paolo M. Triozzi, Rishikesh P. Bhalerao, Mariano Perales

**Affiliations:** ^1^Umeå Plant Science Centre, Department of Forest Genetics and Plant Physiology, Swedish University of Agricultural Sciences, Umeå, Sweden; ^2^Centro de Biotecnología y Genómica de Plantas, Universidad Politécnica de Madrid-Instituto Nacional de Investigación y Tecnología Agraria y Alimentaria, Madrid, Spain

**Keywords:** poplar, adaptive response, cold response, circadian clock, short day, low ambient temperature, bud set, winter dormancy

## Abstract

Boreal and temperate woody perennials are highly adapted to their local climate, which delimits the length of the growing period. Moreover, seasonal control of growth-dormancy cycles impacts tree productivity and geographical distribution. Therefore, traits related to phenology are of great interest to tree breeders and particularly relevant in the context of global warming. The recent application of transcriptional profiling and genetic association studies to poplar species has provided a robust molecular framework for investigating molecules with potential links to phenology. The environment dictates phenology by modulating the expression of endogenous molecular switches, the identities of which are currently under investigation. This review outlines the current knowledge of these molecular switches in poplar and covers several perspectives concerning the environmental control of growth-dormancy cycles. In the process, we highlight certain genetic pathways which are affected by short days, low temperatures and cold-induced signaling.

## Introduction

When the photoperiod falls below the critical day length, poplars undergo growth cessation, culminating in bud set and the acquisition of cold hardiness. The fact that components of light signaling, the circadian clock and orthologs of Arabidopsis flowering time regulators have all been implicated in this stage suggests the interplay between environmental signals and diurnal gene expression in dormancy switch. Thereafter, the sequential induction of ethylene and abscisic acid signaling pathways promotes bud maturation, cessation of meristematic activity and the establishment of dormancy. Once dormant, meristem becomes insensitive to growth-promoting signals. Release from dormancy strongly depends on the accumulation of a defined number of chilling hours, a mechanism for which the molecular basis is unknown. Under spring conditions, the low temperature (LT)-mediated activation of growth promoters reactivates meristem growth. Thus, poplar phenology is controlled by the orchestrated activity of molecular switches following exposure to environmental cues. This mini-review provides insight into the genetic network underlying poplar bud set, dormancy establishment and bud break with a focus on photoperiod- and temperature-dependent signaling. Moreover, we show evidence that bud set is highly sensitive to low ambient temperatures and identify candidate genes that may participate in this response.

## Short Days Promote Acclimation to Winter Conditions During Bud Set

Irrefutable experimental evidence for the short day (SD) requirement to cold acclimation comes from studies of photoperiod-insensitive oat *PHYA* overexpressing poplars Junttila and Kaurin, 1990; Olsen et al., 1997; Welling et al., 2002). When transgenic and wild type plants grown during SDs were subjected to freezing conditions, poplars overexpressing oat *PHYA* did not develop cold hardiness (Olsen et al., 1997; Welling et al., 2002). In contrast, neither transgenic nor wild type poplars grown during long days were able to survive freezing temperatures Junttila and Kaurin, 1990. This indicates that exposure to SDs acclimates poplars to cold weather conditions, and possibly other environmental stresses, which suggests that SDs may activate adaptation pathways.

The transcriptional and metabolomic profiling of poplar shoot apices and stem tissues grown under LD and SD conditions has been used to investigate the molecular signatures underlying SD-driven adaptive responses. The results revealed that SD conditions significantly alter the transcription of certain genes (Rohde et al., 2007; Ruttink et al., 2007; Karlberg et al., 2010; Hoffman et al., 2010; Resman et al., 2010). For example, a reduction in day length affects the transcription of several light signaling- and circadian clock-regulated genes (Ruttink et al., 2007). Moreover, SD also induces genes related to dehydration and cold adaptation, such as *LATE EMBRYOGENESIS-ABUNDANT* (*LEA*), *DEHYDRIN* (*DHN*), *COLD REGULATED GENES* (*COR*), and a set of transcription factors that mediate responses to abiotic stimuli, among others (Ruttink et al., 2007; Karlberg et al., 2010). Continued SD exposure stimulates ethylene signaling and abscisic acid (ABA) hormonal burst (Rohde et al., 2002; Ruttink et al., 2007; Karlberg et al., 2010). Ethylene and ABA are abiotic stress-related phytohormones required for adaptive responses in plants and are also functionally associated to the regulation of many aspects of bud development in poplar and birch (Rohde et al., 2002; Ruonala et al., 2006; Shi and Yang, 2014; Müller and Munné-Bosch, 2015; Tylewicz et al., 2015; Trivedi et al., 2016; Tylewicz et al., 2018). Thus, acclimation to winter conditions is mediated by the temporal orchestration of SD-induced transcriptional responses that partly originate from light signaling and circadian clock dependent pathways. As a result, poplars with modified circadian clock sensitivity showed different tolerance to freezing temperatures (Ibañez et al., 2010).

## Temperature Modulates Bud Set

**FIGURE 1:**
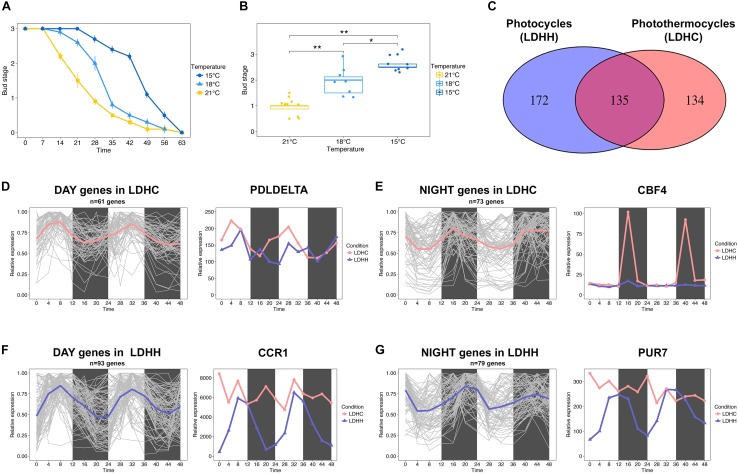
Bud set-associated genes in poplar are sensitive to low ambient temperature. **(A)** Bud set scores for hybrid poplar (*Populus tremula x P. alba*) plants grown under SD and three different temperatures (21, 18, and 15°C) for 9 weeks. **(B)** Comparative analysis of hybrid poplar bud stage progression in plants grown under SD and three different temperatures (21, 18, and 15°C) over 4 weeks. Asterisks denote significant differences between hybrid poplar plant groups (*n* = 8) grown at different temperatures (Kruskal–Wallis test followed by pairwise Wilcox test; ^∗^*P* < 0.05, ^∗∗^*P* < 0.01). **(C)** Venn diagram representing the intersection between genes with SNPs associated with bud set and that show robust diurnal oscillations (cut-off = 0.8) under light/dark cycles (LD) and constant temperature (25°C, HH) or photothermocycles (25°C during the day, 12°C during the night, HC). 172 genes (lilac) show robust diurnal rhythms only under LDHH, 134 genes (pink) show diurnal rhythms only under LDHC and 135 genes (fuchsia) oscillate under both conditions, respectively. **(D,E)** Clusters of genes that show diurnal expression within 0–11 h under LDHC (**D**, mean = pink) and within 12–23 h under LDHC (**E**, mean = pink). The line chart demonstrates how photothermocycles (LDHC) promote diurnal oscillation of poplar PDLDELTA **(D)** and CBF4 genes **(E)**. **(F,G)** Clusters of genes that show diurnal expression within 0–11 h under LDHH (**F**, mean = lilac) and within 12–23 h under LDHH (**G**, mean = lilac). The line chart demonstrates how photothermocycles (LDHC) disrupt the diurnal oscillation of poplar CCR1 **(F)** and PUR7 genes **(G)**.

The effect of temperature on bud set has been poorly investigated at the molecular level. Earlier physiological studies have shown that bud set is a thermosensitive process in trees (reviewed in Tanino et al., 2010). Furthermore, recent phenotyping studies using clonally replicated poplars grown under natural conditions show that the timing of bud set depends on the local climate (Rohde et al., 2011; Evans et al., 2014). Rohde et al. (2011) showed that sub-optimal growth temperatures delay the timing of bud set. Accordingly, we observed that the timing of bud set in hybrid aspen (*Populus tremula x P. alba*) grown under controlled conditions is highly sensitivity to small changes in ambient temperature (Figures 1A,B). Bud set was delayed by approximately 7 days in plants grown at 18°C relative to plants grown at 21°C, and this difference was amplified when the temperature was further reduced to 15°C (Figures 1A,B). This indicates that ambient temperature can impact the SD responses controlling bud set timing. Thus, it can be hypothesized that the activation or repression of thermosensitive genes underlies the regulation of pathways that control bud set. Numerous biological processes are governed by the endogenous clock (Greenham and McClung, 2015); thus, bud set in response to low ambient temperature may additionally be mediated by the modulation of clock-related pathways. For instance, the transcription of cold tolerance genes, e.g., *C-REPEAT BINDING FACTOR (CBF)* genes, follow circadian rhythms in Arabidopsis (Fowler et al., 2005; Dong et al., 2011).

To identify poplar candidate genes that could affect the timing of bud set under low ambient temperature, we investigated how diurnal photothermocycles affect bud set-associated single nucleotide polymorphism (SNP) genes (Mockler et al., 2007; Filichkin et al., 2011; Evans et al., 2014). Particularly, we analyzed the diurnal oscillation (cut-off 0.8) of bud set-associated SNP genes in two different conditions, photothermocycles (LDHC; Day 25°C/Night 12°C) and photocycles (LDHH; Day 25°C/Night 25°C), in *Populus thricocarpa* using the Diurnal database^1^ (Mockler et al., 2007; Filichkin et al., 2011). A total of 134 genes associated with bud set showed robust diurnal transcription patterns over a 48 h period under LDHC conditions but not under constant temperature (LDHH), which suggests that the lower night temperature promotes their rhythmic expression (Figures 1C–E). Within the clusters of genes affected by photothermal cycles, we identified diurnal expression in the poplar ortholog of Arabidopsis *PHOSPHOLIPASE D DELTA* (*PLDDELTA*; Potri.007G060300), which is involved in phospholipid metabolism, freezing tolerance and stomatal closure (Figure 1D; Chen et al., 2008a,b; Distéfano et al., 2012; Uraji et al., 2012). Moreover, the poplar ortholog of *C-REPEAT-BINDING FACTOR 4* (*CBF4*; Potri.012G134100), which mediates the response to decreased temperatures in Arabidopsis (Wang and Hua, 2009), is also induced by cold temperatures in poplar and birch (Figure 1E; Benedict et al., 2006; Welling and Palva, 2008). These examples indicate that the rhythmic expression of genes associated with bud set – stimulated by diurnal oscillations in temperature – may be required for cold acclimation. In contrast, we identified 172 genes with bud set-associated SNPs that showed diurnal transcription patterns under constant temperatures (LDHH) but not under photothermocycles (LDHC). This suggests that reduced night-time temperatures can undermine the rhythmic expression of certain genes (Figures 1C,F,G). Within the clusters of genes with diurnal expression disrupted by photothermocycles, we highlight the poplar ortholog of Arabidopsis *COLD, CIRCADIAN RHYTHM, AND RNA BINDING 1* (*CCR1*; Potri.009G116400) gene (Figure 1F). CCR1 contains an RNA recognition motif (RRM), which promotes alternative splicing that is coupled to degradation by nonsense-mediated decay (NMD) (Schöning et al., 2008). *CCR1* is also regulated by both cold conditions and circadian rhythms in Arabidopsis (Carpenter et al., 1994). Furthermore, we found that the diurnal expression of the poplar ortholog of Arabidopsis *PURIN 7* (*PUR7*; Potri.017G051500), which is required to generate purine dependent cofactors in tissues under high rates of cell division, was disrupted by low night-time temperatures (Senecoff et al., 1996). These examples indicate that the impairment of key, diurnally regulated nucleic acid metabolism processes by decreased night-time temperatures could be important to timely bud set in poplar. Future functional studies will hopefully elucidate the genetic network involved in bud set regulation when poplars are exposed to LTs.

## Cold Disruption of Circadian Clock and Bud Set

The circadian clock creates endogenous, 24 h rhythms to help plants and animals anticipate daily and seasonal environmental changes (Schultz and Kay, 2003). The circadian clock controls physiology, growth and development, as temporal transcriptional profiles have revealed that more than 30% of genes in Arabidopsis and poplar show circadian rhythms (Harmer et al., 2000; Covington et al., 2008; Michael et al., 2008; Hoffman et al., 2010). Recent research has revealed a role for the circadian clock in the genetic network regulating growth-dormancy cycles. It is widely accepted that circadian rhythms play a central role in the photoperiodic mechanism that controls poplar shoot apical growth (reviewed in Triozzi et al., 2018). However, the implication of the biological clock in the control of growth cessation and bud set has recently emerged (Ibañez et al., 2010; Kozarewa et al., 2010; Ding et al., 2018). The circadian rhythms of several pathways involved in growth cessation and bud set can persist 8–10 weeks after exposure to continuous SDs (Triozzi et al., personal communication). This is supported by the finding that the downregulation of poplar clock-related genes *LATE ELONGATED HYPOCOTYL* (*LHY*) and *TIMING OF CAB EXPRESSION 1* (*TOC1*) delays growth cessation and bud set (Ibañez et al., 2010). Furthermore, it has been firstly shown that chestnut clock-related genes display arrhythmic expression under winter conditions (Ramos et al., 2005; Ibañez et al., 2008). Moreover, chestnut and poplar clock-related genes respond to cold temperatures (4°C), showing high and constant expression irrespective of photoperiod (Ramos et al., 2005; Ibañez et al., 2010). It has been suggested that circadian clock disruption may facilitate bud set under cold temperatures (Johansson et al., 2015). Accordingly, cold-induced disruption of circadian rhythms caused wide transcriptional rearrangement of cold response genes in Arabidopsis (Bieniawska et al., 2008). Additionally, when exposed to freezing temperatures, *LHY*-RNAi poplars showed far more severe stem injuries than control plants. This indicates that the circadian clock is pivotal in the development of cold hardiness during bud set (Ibañez et al., 2010). Nevertheless, the functional implications of cold-induced disruption of clock-regulated pathways during bud set needs further investigation.

## Dormancy Establishment

**FIGURE 2:**
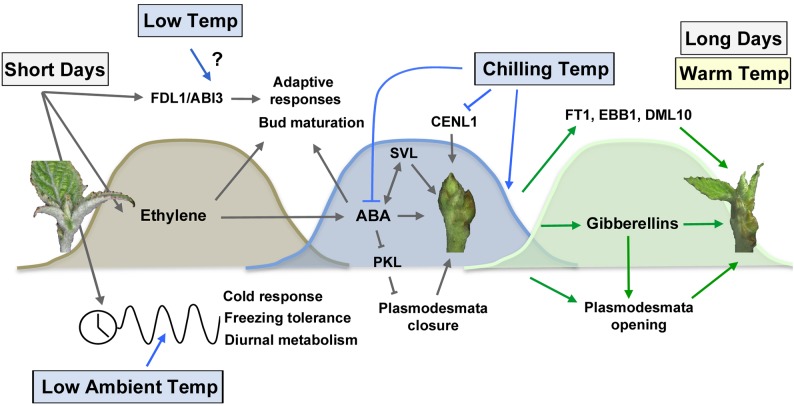
Photoperiodic and temperature control of bud set, dormancy establishment and bud break. Short days promote bud set via activation of *Flowering locus D1* (*FLD1*) and *Abscisic acid insensitive 3* (*ABI3*) (Tylewicz et al., 2015), the ethylene and ABA pathways (Rohde et al., 2002; Ruonala et al., 2006) as well as the circadian clock pathway (Hoffman et al., 2010; Ibañez et al., 2010). The timing of bud set is sensitive to low ambient temperature, which alters the expression of the circadian pathway (Figure 1). ABA plays an essential role in dormancy establishment by promoting plasmodesmata closure via PKL repression (Tylewicz et al., 2018) and activating SVL growth repressive gene (Singh et al., 2018). Increased exposure to chilling temperatures is necessary to the downregulation of ABA and degradation of growth repressors such as CENL1 (Karlberg et al., 2010; Mohamed et al., 2010; Tylewicz et al., 2018). Dormancy release increases gibberellin levels (Rinne et al., 2011), mediates plasmodesmata opening (Tylewicz et al., 2018) and activates bud break-promoting factors such as FT1 (Hsu et al., 2011; Rinne et al., 2011), EBB1 (Yordanov et al., 2014), and DML10 (Conde et al., 2017). Long days and warm temperatures restore shoot apical growth.

Prolonged SD exposure after growth cessation and bud set results in dormancy establishment in various plants (Heide, 1974; Espinosa-Ruiz et al., 2004; Ruttink et al., 2007; Maurya and Bhalerao, 2017). However, until recently, our knowledge of the molecular basis of bud dormancy was rudimentary. Conclusive evidences for dormancy establishment has come from studies on plant hormones ethylene and ABA, both of which are required for apical bud formation (Figure 2). For example, ethylene-insensitive dominant mutant *etr1-1* birch plants failed to develop apical buds under SD conditions (Ruonala et al., 2006). Ethylene has also been suggested to participate in dormancy induction in plants such as leafy spurge and *Chrysanthemum* (Sumitomo et al., 2008; Doğramacı et al., 2013. Nevertheless, genetic evidences for how ethylene affects bud dormancy are missing.

Abscisic acid levels increase following SD exposure (Ruttink et al., 2007; Karlberg et al., 2010). Interestingly, the apical buds of *etr1-1* mutant birches failed to accumulate ABA under SD conditions, which may suggest that defects in these plants may stem from the inability to increase ABA levels. Moreover, under SD conditions, *ABI3oe* plants display apical buds with defects during bud maturation (Rohde et al., 2002; Ruttink et al., 2007). Although poplar *ABI3* has not been demonstrated to be ABA responsive or contribute to the ABA response *per se*, this finding may suggest a role for ABA, or at least ABI3, in bud maturation. Other research suggests that molecules associated with the opening and closing of plasmodesmata are involved in dormancy establishment (Rinne et al., 2011). It has been proposed that plasmodesmata closure in shoot apical meristem (SAM) is important for dormancy establishment (Rinne et al., 2011; Singh et al., 2017). Growth-promoting signals such a florigen move symplastically through plasmodesmata to SAM to promote growth under appropriate conditions, yet molecular and genetic evidence for this mechanism was only recently published (Singh et al., 2017; Tylewicz et al., 2018).

Abscisic acid-insensitive hybrid aspen plants overexpressing the dominant negative *abi1-1* allele failed to establish dormancy under short photoperiods (SPs) (Tylewicz et al., 2018). Both WT and *abi1-1* plants underwent growth cessation and apical bud formation when grown under SPs. However, when transferred from 11 weeks of SP to growth-promoting long photoperiods (LPs), WT plants did not start growing, which suggests that these plants were in a dormant state. However, *abi1-1* lines restarted growth 11–15 days after being transferred to LPs. The transcriptomic analysis of WT and *abi1-1* plants after 0, 6, and 10 weeks of SPs suggests that 1000s of genes are differentially regulated. Interestingly, many of these genes are associated with plasmodesmata closure and opening. Plasmodesmata closure-related genes such as *GERMIN-LIKE 10*, *REMORIN-LIKE 1* and *2* and *CALLOSE SYNTHASE 1* were upregulated in the apices of WT plants whereas genes related to plasmodesmata opening, such as *GH17_39*, were downregulated. The transcript levels of these genes differed between *abi1-1* and WT plants. Microscopic analyses of WT and *abi1-1* plants suggest that 83 and less than 3% of plasmodesmata in these plants, respectively, were closed after 10W of SPs (Tylewicz et al., 2018). Flowering Locus T (FT) protein acts as a mobile signal that can move from leaves to apex (Corbesier et al., 2007) and promotes growth of poplar trees even under SP (Böhlenius et al., 2006). Furthermore, when rootstocks of Flowering Locus T1 (FT1) plants were grafted to the scions of 10W SP grown WT and *abi1-1* plants, it was able to activate the growth of *abi1-1* plants even in SP conditions but not in WT scions.

Many of the transcripts associated with the plasmodesmata closure and opening also responded to the SPs (Tylewicz et al., 2018). Overexpression of plasmodesmata-located protein 1 (PDLP1) in *abi1-1* plants leads to dormancy establishment, as *abi1-1*/*PDLP1*oe plants transferred from SPs to LPs did not show growth reactivation. Certain genes involved in chromatin remodeling, such as *FERTILIZATION INDEPENDENT ENDOSPERM (FIE)* and *PICKLE (PKL)*, have been shown to be upregulated after SD exposure (Ruttink et al., 2007). FIE is a component of the polycomb repressive complex 2 (PRC2), while PKL is its antagonist. PRC2 plays important roles in keeping the genes in the repressed state (Margueron and Reinberg, 2011). Transcript level of *PKL* was upregulated in *abi1-1* plants, and its downregulation in *abi1-1* background developed dormancy by closing plasmodesmata (Tylewicz et al., 2018).

Taken together, these results suggest that SP influences ABA levels, which then differentially regulate plasmodesmata opening and closure-related genes to induce dormancy. Once the plasmodesmata are closed, growth-promoting signals cannot enter the SAM due to the formation of dormancy sphincters through callose deposition in plasmodesmata.

## Dormancy Release and Bud Burst

As mentioned earlier, dormancy establishment makes the SAM impermeable to growth-promoting signals, even under LD conditions. Release from dormancy requires that buds are exposed to LT for a prolonged period (Saure, 1985; Hannerz et al., 2003; Brunner et al., 2014; Fu et al., 2015). How temperature regulates dormancy release remains a poorly studied topic. Interestingly, the exposure of chilled buds to warm temperature is sufficient to induce dormancy release and subsequent bud burst. By monitoring the dormancy release time in plants with altered basal level of certain genes, we can anticipate their role in this process. Since plasmodesmata closure is required for dormancy establishment (Tylewicz et al., 2018), their opening could be expected to result in dormancy release. It has been suggested that LT treatment leads to the opening of plasmodesmata (Rinne et al., 2011), but this does not have proper experimental proof until now. However, some of the genes needed for the removal of plasmodesmatal dormancy sphincters by degrading the deposited callose, such as 1,3-β-glucanase (glucan hydrolase family 17 [GH17]), are upregulated after chilling treatment (Rinne et al., 2011).

Gibberellic acids (GAs) and FT are positive regulators of growth; as such, LT induces the transcription of *FT1* and genes implicated in GA metabolism, those that encode members of the GA3 and GA20 oxidases (Rinne et al., 2011). GAs may promote *GH17s* expression to reopen the plasmodesmata and thus restart the symplastic, growth-promoting cell-to-cell signaling within the SAM (Figure 2). However, genetic and experimental proof of this mechanism is still lacking. *CONSTANS (CO)* expression was shown to be upregulated almost threefold after 2 weeks of chilling. This suggests that the CO/FT module may be initiated by LT, yet *CO* expression remains at a similar level throughout the cold period and is only further enhanced when chilled buds sense LDs and warmer temperatures (Rinne et al., 2011). However, the expression of *CENL1/TFL1* (*CENTRORADIALIS-LIKE1/TERMINAL FLOWER 1*), a negative regulator of growth, remains at low levels in chilled buds throughout the LT period (Figure 2; Rinne et al., 2011). Experiments with *CENL1oe* and *CENL*-RNAi lines showed delayed and early bud burst, respectively, relative to wild type plants (Mohamed et al., 2010). This suggests that CENL1 represses dormancy release and bud burst. In chilled buds, CENL1 expression is only upregulated once the buds are transferred to warmer temperatures.

The process of dormancy release in trees is comparable to vernalization in Arabidopsis (Chouard, 1960). Many of the MAD-box transcription factors – termed DORMANCY ASSOCIATED MADS-BOX (DAM) – are known to be induced in the dormant buds of many plants, and are similar to short vegetative protein (SVP) and AGAMOUS-LIKE (AGL) transcription factors which control flowering in Arabidopsis (Ríos et al., 2014). Similar to *Flowering Locus C* (*FLC*) in Arabidopsis, these *DAM* genes are also epigenetically regulated by cold conditions. Cold-induced epigenetic silencing of floral repressor *FLC* is required during vernalization to induce flowering in Arabidopsis (Amasino, 2004; Bastow et al., 2004). The finding that expression of *DAM* genes goes down after cold treatment suggests that these genes are repressors of dormancy release (Shim et al., 2014; Howe et al., 2015). However, the exact roles of *DAM* genes can only be elucidated through future genetic studies.

Like dormancy release, our knowledge about bud burst is also very limited in perennial plants. A large number of genes are differentially regulated during dormancy release (Shim et al., 2014; Howe et al., 2015), but we do not have enough genetic evidence to conclusively describe their roles in dormancy release. Very recently, a genetic network mediating the dormancy release and bud break has been described (Singh et al., 2018). This study demonstrated that short vegetative protein-like (SVL), a tree ortholog of Arabidopsis SVP, acts as a central regulator of dormancy release and bud burst in poplar. It negatively regulates dormancy release and bud burst by promoting and repressing the expressions of negative and positive regulators of growth, respectively, after cold treatment (Singh et al., 2018). The expression of *Early Bud-Break 1* (*EBB1*), a putative APETALA2/Ethylene responsive factor, also increases after cold periods during dormancy release and before bud burst. However, *EBB1* transcripts are undetectable during the dormancy period. Transgenic lines with *EBB1* overexpression and downregulation show early and delayed bud burst relative to wild type plants, respectively. This suggests that EBB1 is a positive regulator of bud burst (Figure 2; Yordanov et al., 2014).

## Concluding Remarks

The effect of temperature on tree phenology is an important topic in the context of climate change. Extended seasonal growth and shifts in latitudinal distribution demonstrate how plants are already adapting to increasing global temperatures. Although previous research has attempted to elucidate how temperature influences growth cessation and bud set in trees, very little is still known about the molecular mechanisms involved in these phenomena. For this reason, understanding how environmentally sensitive molecular switches regulate the perception and transduction of temperature signals in woody plants will be crucial to predicting how plants will adapt to warmer environments and designing appropriate breeding programs for this scenario.

## Author Contributions

PT and MP analyzed the data. JM, PT, RB, and MP contributed to the writing of the manuscript.

## Conflict of Interest Statement

The authors declare that the research was conducted in the absence of any commercial or financial relationships that could be construed as a potential conflict of interest.

## Footnotes

1 http://diurnal.mocklerlab.org

## References

[B1] Amasino R. (2004). Vernalization, competence, and the epigenetic memory of winter. *Plant Cell* 16 2553–2559. 10.1105/tpc.104.161070 15466409 PMC520954

[B2] Bastow R. Mylne J. S. Lister C. Lippman Z. Martienssen R. A. Dean C. (2004). Vernalization requires epigenetic silencing of FLC by histone methylation. *Nature* 427 164–167. 10.1038/nature02269 14712277

[B3] Benedict C. Skinner J. S. Meng R. Chang Y. Bhalerao R. Huner N. P. A. (2006). The CBF1-dependent low temperature signalling pathway, regulon and increase in freeze tolerance are conserved in Populus spp. *Plant Cell Environ.* 29 1259–1272. 10.1111/j.1365-3040.2006.01505.x 17080948

[B4] Bieniawska Z. Espinoza C. Schlereth A. Sulpice R. Hincha D. K. Hannah M. A. (2008). Disruption of the Arabidopsis circadian clock is responsible for extensive variation in the cold-responsive transcriptome. *Plant Physiol.* 147 263–279. 10.1104/pp.108.118059 18375597 PMC2330297

[B5] Böhlenius H. Huang T. Charbonnel-Campaa L. Brunner A. M. Jansson S. Strauss S. H. (2006). CO/FT regulatory module controls timing of flowering and seasonal growth cessation in trees. *Science* 312 1040–1043. 10.1126/science.1126038 16675663

[B6] Brunner A. M. Evans L. M. Hsu C. Y. Sheng X. (2014). Vernalization and the chilling requirement to exit bud dormancy: shared or separate regulation? *Front. Plant Sci.* 5:732. 10.3389/fpls.2014.00732 25566302 PMC4269124

[B7] Carpenter C. D. Kreps J. A. Simon A. E. (1994). Genes encoding glycine-rich *Arabidopsis thaliana* proteins with RNA-binding motifs are influenced by cold treatment and an endogenous circadian rhythm. *Plant Physiol.* 104 1015–1025. 10.1104/pp.104.3.1015 7513083 PMC160700

[B8] Chen Q. F. Xiao S. Chye M. L. (2008a). Arabidopsis ACBP6 is an acyl-CoA-binding protein associated with phospholipid metabolism. *Plant Signal. Behav.* 3 1019–1020. 10.4161/psb.6762 19704440 PMC2633763

[B9] Chen Q. F. Xiao S. Chye M. L. (2008b). Overexpression of the Arabidopsis 10-kilodalton Acyl-coenzyme a-binding protein acbp6 enhances freezing tolerance. *Plant Physiol.* 148 304–315. 10.1104/pp.108.123331 18621979 PMC2528132

[B10] Chouard P. (1960). Vernalization and its relations to dormancy. *Annu. Rev. Plant Physiol.* 11 191–238. 10.1146/annurev.pp.11.060160.001203 14737827

[B11] Conde D. Le Gac A. L. Perales M. Dervinis C. Kirst M. Maury S. (2017). Chilling-responsive demeter-like DNA demethylase mediates in poplar bud break. *Plant Cell Environ.* 40 2236–2249. 10.1111/pce.13019 28707409

[B12] Corbesier L. Vincent C. Jang S. Fornara F. Fan Q. Searle I. (2007). FT protein movement contributes to long-distance signaling in floral induction of Arabidopsis. *Science* 316 1030–1033. 10.1126/science.1141752 17446353

[B13] Covington M. F. Maloof J. N. Straume M. Kay S. A. Harmer S. L. (2008). Global transcriptome analysis reveals circadian regulation of key pathways in plant growth and development. *Genome Biol.* 9:R130. 10.1186/gb-2008-9-8-130 18710561 PMC2575520

[B14] Ding J. Böhlenius H. Rühl M. G. Chen P. Sane S. Zambrano J. A. (2018). Gigantea-like genes control seasonal growth cessation in populus. *New Phytol.* 218 1491–1503. 10.1111/nph.15087 29532940

[B15] Distéfano A. M. Scuffi D. García-Mata C. Lamattina L. Laxalt A. M. (2012). Phospholipase Dδ is involved in nitric oxide-induced stomatal closure. *Planta* 236 1899–1907. 10.1007/s00425-012-1745-4 22932846

[B16] Doğramacı M. Foley M. E. Chao W. S. Christoffers M. J. Anderson J. V. (2013). Induction of endodormancy in crown buds of leafy spurge (*Euphorbia esula* L.) implicates a role for ethylene and cross-talk between photoperiod and temperature. *Plant Mol. Biol.* 81 577–593. 10.1007/s11103-013-0026-3 23436173

[B17] Dong M. A. Farre E. M. Thomashow M. F. (2011). Circadian clock-associated 1 and late elongated hypocotyl regulate expression of the c-repeat binding factor (cbf) pathway in Arabidopsis. *Proc. Natl. Acad. Sci. U.S.A.* 108 7241–7246. 10.1073/pnas.1103741108 21471455 PMC3084081

[B18] Espinosa-Ruiz A. Saxena S. Schmidt J. Mellerowicz E. Miskolczi P. Bako L. (2004). Differential stage-specific regulation of cyclin-dependent kinases during cambial dormancy in hybrid aspen. *Plant J.* 38 603–615. 10.1111/j.1365-313X.2004.02070.x 15125767

[B19] Evans L. M. Slavov G. T. Rodgers-Melnick E. Martin J. Ranjan P. Muchero W. (2014). Population genomics of Populus trichocarpa identifies signatures of selection and adaptive trait associations. *Nat. Genet.* 46 1089–1096. 10.1038/ng.3075 25151358

[B20] Filichkin S. A. Breton G. Priest H. D. Dharmawardhana P. Jaiswal P. Fox S. E. (2011). Global profiling of rice and poplar transcriptomes highlights key conserved Circadian-controlled pathways and cis-regulatory modules. *PLoS One* 6:e16907. 10.1371/journal.pone.0016907 21694767 PMC3111414

[B21] Fowler S. G. Cook D. Thomashow M. F. (2005). Low temperature induction of Arabidopsis CBF1,2, and 3 is gated by the circadian clock. *Plant Physiol.* 137 961–968. 10.1104/pp.104.058354 15728337 PMC1065397

[B22] Fu Y. H. Piao S. Vitasse Y. Zhao H. Boeck H. J. D. Liu Q. (2015). Increased heat requirement for leaf flushing in temperate woody species over 1980–2012: effects of chilling, precipitation and insolation. *Global Change Biol.* 21 2687–2697. 10.1111/gcb.12863 25580596

[B23] Greenham K. McClung C. R. (2015). Integrating circadian dynamics with physiological processes in plants. *Nat. Rev. Genet.* 16 598–610. 10.1038/nrg3976 26370901

[B24] Hannerz M. Ekberg I. Norell L. (2003). Variation in chilling requirements for completing bud rest between provenances of Norway spruce. *Silvae Genet.* 52 161–168.

[B25] Harmer S. L. Hogenesch J. B. Straume M. Chang H. S. Han B. Zhu T. (2000). Orchestrated transcription of key pathways in Arabidopsis by the circadian clock. *Science* 290 2110–2113. 10.1126/science.290.5499.2110 11118138

[B26] Heide O. M. (1974). Growth and dormancy in Norway spruce ecotypes (*Picea abies*) I. Interaction of photoperiod and temperature. *Physiol. Plant.* 30 1–12. 10.1111/j.1399-3054.1974.tb04983.x

[B27] Hoffman D. E. Jonsson P. Bylesjö M. Trygg J. Antti H. Eriksson M. E. (2010). Changes in diurnal patterns within the Populus transcriptome and metabolome in response to photoperiod variation. *Plant Cell Environ.* 33 1298–1313. 10.1111/j.1365-3040.2010.02148.x 20302601

[B28] Howe G. T. Horvath D. P. Dharmawardhana P. Priest H. D. Mockler T. C. Strauss S. H. (2015). Extensive transcriptome changes during natural onset and release of vegetative bud dormancy in Populus. *Front. Plant Sci.* 6:989. 10.3389/fpls.2015.00989 26734012 PMC4681841

[B29] Hsu C. Y. Adams J. P. Kim H. No K. Ma C. Strauss S. H. (2011). Flowering locus t duplication coordinates reproductive and vegetative growth in perennial poplar. *Proc. Natl. Acad. Sci. U.S.A.* 108 10756–10761. 10.1073/pnas.1104713108 21653885 PMC3127867

[B30] Ibañez C. Kozarewa I. Johansson M. Ogren E. Rohde A. Eriksson M. E. (2010). Circadian clock components regulate entry and affect exit of seasonal dormancy as well as winter hardiness in Populus trees. *Plant Physiol.* 153 1823–1833. 10.1104/pp.110.158220 20530613 PMC2923903

[B31] Ibañez C. Ramos A. Acebo P. Contreras A. Casado R. Allona I. (2008). Overall alteration of circadian clock gene expression in the chestnut cold response. *PLoS One* 3:e3567. 10.1371/journal.pone.0003567 18958171 PMC2569414

[B32] Johansson M. Ramos-Saìnchez J. M. Conde D. IbaìnÞez C. Takata N. Allona I. (2015). “Role of the circadian clock in cold acclimation and winter dormancy in perennial plants,” in *Advances in Plant Dormancy*, ed. Anderson J. V. (Basel: Springer International Publishing), 51–74.

[B33] Junttila O. Kaurin Å (1990). Environmental control of cold acclimation in salix pentandra. *Scand. J. For. Res.* 5 195–204. 10.1080/02827589009382605

[B34] Karlberg A. Englund M. Petterle A. Molnar G. Sjödin A. Bako L. (2010). Analysis of global changes in gene expression during activity-dormancy cycle in hybrid aspen apex. *Plant Biotechnol.* 27 1–16. 10.5511/plantbiotechnology.27.1

[B35] Kozarewa I. Ibanez C. Johansson M. (2010). Alteration of PHYA expression change circadian rhythms and timing of bud set in Populus. *Plant Mol. Biol.* 73 143–156. 10.1007/s11103-010-9619-2 20229130

[B36] Margueron R. Reinberg D. (2011). The polycomb complex PRC2 and its mark in life. *Nature* 469 343–349. 10.1038/nature09784 21248841 PMC3760771

[B37] Maurya J. P. Bhalerao R. P. (2017). Photoperiod- and temperature-mediated control of growth cessation and dormancy in trees: a molecular perspective. *Ann. Bot.* 120 351–360. 10.1093/aob/mcx061 28605491 PMC5591416

[B38] Michael T. P. Mockler T. C. Breton G. McEntee C. Byer A. Trout J. D. (2008). Network discovery pipeline elucidates conserved time-of-day-specific cis-regulatory modules. *PLoS Genet.* 4:e14. 10.1371/journal.pgen.0040014 18248097 PMC2222925

[B39] Mockler T. C. Michael T. P. Priest H. D. Shen R. Sullivan C. M. Givan S. A. (2007). The diurnal project: diurnal and circadian expression profiling, model-based pattern matching, and promoter analysis. *Cold Spring Harb. Symp. Quant. Biol.* 72 353–363. 10.1101/sqb.2007.72.006 18419293

[B40] Mohamed R. Wang C. T. Ma C. Shevchenko O. Dye S. J. Puzey J. R. (2010). Populus CEN/TFL1 regulates first onset of flowering, axillary meristem identity and dormancy release in Populus. *Plant J.* 62 674–688. 10.1111/j.1365-313X.2010.04185.x 20202169

[B41] Müller M. Munné-Bosch S. (2015). Ethylene response factors: a key regulatory hub in hormone and stress signaling. *Plant Physiol.* 169 32–41. 10.1104/pp.15.00677 26103991 PMC4577411

[B42] Olsen J. E. Junttila O. Nilsen J. Eriksson M. E. Martinussen I. Olsson O. (1997). Ectopic expression of oat phytochrome A in hybrid aspen changes critical daylength for growth and prevents cold acclimatization. *Plant J.* 12 1339–1350. 10.1046/j.1365-313x.1997.12061339.x

[B43] Ramos A. Pérez-Solís E. Ibáñez C. Casado R. Collada C. Gómez L. (2005). Winter disruption of the circadian clock in chestnut. *Proc. Natl. Acad. Sci. U.S.A.* 102 7037–7042. 10.1073/pnas.0408549102 15860586 PMC1100755

[B44] Resman L. Howe G. Jonsen D. Englund M. Druart N. Schrader J. (2010). Components acting downstream of short day perception regulate differential cessation of cambial activity and associated responses in early and late clones of hybrid poplar. *Plant Physiol.* 154 1294–1303. 10.1104/pp.110.163907 20847139 PMC2971607

[B45] Rinne P. L. Welling A. Vahala J. Ripel L. Ruonala R. Kangasjarvi J. (2011). Chilling of dormant buds hyperinduces flowering locus t and recruits GA-inducible 1,3-betaglucanases to reopen signal conduits and release dormancy in Populus. *Plant Cell* 23 130–146. 10.1105/tpc.110.081307 21282527 PMC3051240

[B46] Ríos G. Leida C. Conejero A. Badenes M. L. (2014). Epigenetic regulation of bud dormancy events in perennial plants. *Front. Plant Sci.* 5:247. 10.3389/fpls.2014.00247 24917873 PMC4042555

[B47] Rohde A. Bastien C. Boerjan W. (2011). Temperature signals contribute to the timing of photoperiodic growth cessation and bud set in poplar. *Tree Physiol.* 31 472–482. 10.1093/treephys/tpr038 21636689

[B48] Rohde A. Prinsen E. De Rycke R. Engler G. Van Montagu M. Boerjan W. (2002). PtABI3 impinges on the growth and differentiation of embryonic leaves during bud set in poplar. *Plant Cell* 14 1885–1901. 10.1105/tpc.003186 12172029 PMC151472

[B49] Rohde A. Ruttink T. Hostyn V. Sterck L. Van Driessche K. Boerjan W. (2007). Gene expression during the induction, maintenance, and release of dormancy in apical buds of poplar. *J. Exp. Bot.* 58 4047–4060. 10.1093/jxb/erm261 18039739

[B50] Ruonala R. Rinne P. L. H. Baghour M. Moritz T. Tuominen H. Kangasjärvi J. (2006). Transitions in the functioning of the shoot apical meristem in birch (*Betula pendula*) involve ethylene. *Plant J.* 46 628–640. 10.1111/j.1365-313X.2006.02722.x 16640599

[B51] Ruttink T. Arend M. Morreel K. Storme V. Rombauts S. Fromm J. (2007). A molecular timetable for apical bud formation and dormancy induction in poplar. *Plant Cell* 19 2370–2390. 10.1105/tpc.107.052811 17693531 PMC2002631

[B52] Saure M. (1985). Dormancy release in deciduous fruit trees. *Hortic. Rev.* 7 239–300. 10.1002/9781118060735.ch6

[B53] Schöning J. C. Streitner C. Meyer I. M. Gao Y. Staiger D. (2008). Reciprocal regulation of glycine-rich RNA-binding proteins via an interlocked feedback loop coupling alternative splicing to nonsense-mediated decay in Arabidopsis. *Nucleic Acids Res.* 36 6977–6987. 10.1093/nar/gkn847 18987006 PMC2602770

[B54] Schultz T. F. Kay S. A. (2003). Circadian clocks in daily and seasonal control of development. *Science* 301 326–328. 10.1126/science.1085935 12869749

[B55] Senecoff J. F. McKinney E. C. Meagher R. B. (1996). De novo purine synthesis in *Arabidopsis thaliana*. II. The PUR7 gene encoding 5’-phosphoribosyl-4-(N-succinocarboxamide)-5-aminoimidazole synthetase is expressed in rapidly dividing tissues. *Plant Physiol.* 112 905–917. 10.1104/pp.112.3.905 8938402 PMC158018

[B56] Shi Y. Yang S. (2014). “ABA regulation of the cold stress response in plants,” in *Abscisic Acid: Metabolism, Transport and Signaling*, ed. Zhang D. P. (Dordrecht: Springer), 337–363. 10.1007/978-94-017-9424-4_17

[B57] Shim D. Ko J. H. Kim W. C. Wang Q. Keathley D. E. Han K. H. (2014). A molecular framework for seasonal growth–dormancy regulation in perennial plants. *Hortic. Res.* 1:14059. 10.1038/hortres.2014.59 26504555 PMC4591672

[B58] Singh R. K. Maurya J. P. Azeez A. Miskolczi P. Tylewicz S. Stojkoviè K. (2018). A genetic network mediating the control of bud break in hybrid aspen. *Nat. Commun.* 9:4173. 10.1038/s41467-018-06696-y 30301891 PMC6177393

[B59] Singh R. K. Svystun T. AlDahmash B. Jönsson A. M. Bhalerao R. P. (2017). Photoperiod- and temperature-mediated control of phenology in trees – a molecular perspective. *New Phytol.* 213 511–524. 10.1111/nph.14346 27901272

[B60] Sumitomo K. Narumi T. Satoh S. Hisamatsu T. (2008). Involvement of the ethylene response pathway in dormancy induction in chrysanthemum. *J. Exp. Bot.* 59 4075–4082. 10.1093/jxb/ern247 18952907 PMC2639020

[B61] Tanino K. K. Kalcsits L. Silim S. Kendall E. Gray G. R. (2010). Temperature-driven plasticity in growth cessation and dormancy development in deciduous woody plants: a working hypothesis suggesting how molecular and cellular function is affected by temperature during dormancy induction. *Plant Mol. Biol.* 73 49–65. 10.1007/s11103-010-9610-y 20191309

[B62] Triozzi P. M. Ramos-Sánchez J. M. Hernández-Verdeja T. Moreno-Cortés A. Allona I. Perales M. (2018). Photoperiodic control of shoot apical growth in poplar. *Front. Plant Sci.* 9:1030. 10.3389/fpls.2018.01030 30057588 PMC6053638

[B63] Trivedi D. K. Gill S. S. Tuteja N. (2016). “Abscisic acid (ABA): biosynthesis, regulation, and role in abiotic stress tolerance,” in *Abiotic Stress Response in Plants*, eds Tuteja N. Gill S. S. (Hoboken, NJ: Wiley), 10.1002/9783527694570.ch15

[B64] Tylewicz S. Petterle A. Marttila S. Miskolczi P. Azeez A. Singh R. K. (2018). Photoperiodic control of seasonal growth is mediated by ABA acting on cell-cell communication. *Science* 360 212–215. 10.1126/science.aan8576 29519919

[B65] Tylewicz S. Tsuji H. Miskolczi P. Petterle A. Azeez A. Jonsson K. (2015). Dual role of tree florigen activation complex component FD in photoperiodic growth control and adaptive response pathways. *Proc. Natl. Acad. Sci. U.S.A.* 112 3140–3145. 10.1073/pnas.1423440112 25713384 PMC4364234

[B66] Uraji M. Katagiri T. Okuma E. Ye W. Hossain M. A. Masuda C. (2012). Cooperative function of pld and pld 1 in abscisic acid-induced stomatal closure in Arabidopsis. *Plant Physiol.* 159 450–460. 10.1104/pp.112.195578 22392280 PMC3375977

[B67] Wang Y. Hua J. (2009). A moderate decrease in temperature induces COR15a expression through the CBF signaling cascade and enhances freezing tolerance. *Plant J.* 60 340–349. 10.1111/j.1365-313X.2009.03959.x 19563440

[B68] Welling A. Moritz T. Palva E. T. Junttila O. (2002). Independent activation of cold acclimation by low temperature and short photoperiod in hybrid aspen. *Plant Physiol.* 129 1633–1641. 10.1104/pp.003814 12177476 PMC166751

[B69] Welling A. Palva E. T. (2008). Involvement of CBF transcription factors in winter hardiness in birch. *Plant Physiol.* 147 1199–1211. 10.1104/pp.108.117812 18467468 PMC2442524

[B70] Yordanov Y. S. Ma C. Strauss S. H. Busov V. B. (2014). Early bud-break 1 (EBB1) is a regulator of release from seasonal dormancy in poplar trees. *Proc. Natl. Acad. Sci. U.S.A.* 111 10001–10006. 10.1073/pnas.1405621111 24951507 PMC4103365

